# Assessment of blood fluidity and the development of the microchannel method for preventive medicine and disease activity evaluation

**DOI:** 10.20407/fmj.2025-048

**Published:** 2026-05-14

**Authors:** Tatsushi Kimura

**Affiliations:** Department of Global Early Childhood Education, School of Early Childhood Education and Care, Ohkagakuen University, Toyoake, Aichi, Japan

**Keywords:** Hemorheology, Blood fluidity, Exercise, Microchannel method

## Abstract

Hemorheology deals with the flow properties of blood. Research has primarily focused on analyzing the state of blood flow and the characteristics of blood in the microcirculation, such as in capillaries. In studies on blood fluidity, the ektacytometry method, which uses a laser-assisted optical rotational cell analyzer, has been the mainstream approach. In contrast, the microchannel method, which uses a microchannel array flow analyzer, was developed in Japan, and is characterized by the ability to analyze samples without any pretreatment and visualize the flow of blood under conditions that closely approximate physiological reality. This review focuses on the microchannel method and provides an overview of blood fluidity, covering the fundamentals of analytical methods, diurnal variations in blood fluidity, factors that impair blood fluidity, exercise-induced disturbances under high-intensity conditions, and the prevention of exercise-induced impairment of blood fluidity.

## Introduction

In hemorheology, it is essential to examine the various factors that influence blood fluidity, including blood viscosity, red blood cell (RBC) deformability, and cellular aggregability, from different perspectives. The Fåhraeus–Lindqvist effect,^1^ a vessel diameter-dependent change in apparent viscosity, and the Fåhraeus effect,^2^ a reduction in the RBC volume as measured by in-tube hematocrit (Hct), are central determinants of intravascular blood fluidity. Blood viscosity exhibits non-Newtonian behavior that varies according to the magnitude and rate of externally applied force, and blood flow is highly dependent on the Hct and plasma viscosity. In addition, RBCs possess intrinsic deformability, and RBCs and platelets exhibit aggregability, whereas WBCs demonstrate adhesive and migratory behaviors.

Instruments used to measure blood fluidity quantify specific aspects of these diverse determinants. A laser-assisted optical rotational cell analyzer (LORRCA; RR Mechatronics B.V.) quantifies RBC deformability via ektacytometry, whereas a RheoScan (RheoMeditech Inc.) analyzer evaluates RBC aggregability using syllectometry. Some models can measure deformability and aggregability, all of which rely on suspension-based systems. In contrast, microchannel methods, such as the microchannel array flow analyzer (MC-FAN; OPTIMA Inc.), allow whole blood to pass through parallel capillary-mimicking channels and evaluate blood fluidity based on passage time. In contrast to LORRCA and RheoScan instruments, the MC-FAN system integrates multiple factors, including RBC deformability, RBC aggregation, leukocyte and platelet obstruction, and plasma viscosity, to provide an overall evaluation. In contrast to ektacytometry and syllectometry, which quantify surrogate indices of individual physical properties, the microchannel method captures the behavior of blood flow within a microcirculatory network, thereby allowing different research questions to be addressed. However, the measurement conditions for MC-FAN systems remain less standardized than those of other techniques. Table 1 summarizes the main instruments used to assess blood fluidity.

## Methods for analyzing blood rheology

### Viscometry

Viscometry measures the torque generated at a constant shear rate between a cone and plate using whole blood, plasma, or resuspended RBCs to determine the apparent viscosity of blood or plasma. According to the International Society for Clinical Hemorheology (ISCH) guidelines,^3^ the key reporting items include: (i) blood sampling and anticoagulation methods, (ii) temperature control (37°C recommended), (iii) standardization of shear history, (iv) minimization of sampling-to-measurement time, and (v) concurrent reporting of Hct and plasma viscosity. Because viscosity values differ according to the type of viscometer (parallel plate, capillary, or cone–plate), an explicit description of the device, procedure, and calibration is essential.^4^

The parallel-plate system^5^ is a rotational rheometer method in which one of two horizontal plates rotates. The viscosity (η) is calculated as η=τ/γ̇, where τ is the shear stress derived from the torque, and γ̇ is the shear rate at a known angular velocity. The capillary system^6^ continuously records pressure or mass signals as blood flows through a capillary tube that is, for example, U-shaped, reconstructing the shear rate and viscosity simultaneously from fluid-dynamic equations using a scanning approach. The cone–plate system^7^ rotates a small-angle cone over a flat plate to create a theoretically uniform shear field (constant γ̇), enabling the acquisition of viscosity–shear-rate flow curves. Blood behaves as a non-Newtonian shear-thinning fluid because of RBC aggregation at low shear and RBC deformation at high shear rates.

### Ektacytometry

Under shear flow, RBCs elongate and become elliptical, and this deformation is quantified using laser diffraction patterns. The elongation index (EI) is calculated from the major (a) and minor (b) axes of the ellipse as EI=(a–b)/(a+b), producing an EI–shear stress (SS) curve.^8^ The ISCH guidelines^3^ emphasize temperature control at 37°C, minimization of sampling-to-measurement intervals, and clear documentation of anticoagulants. As the data reflect population averages, a small fraction of rigid cells may not be detected sensitively.

Typically, the suspension medium is a high-molecular-weight polyvinylpyrrolidone (PVP, ~360 kDa) adjusted to an isotonic solution with a viscosity of approximately 26–30 mPa·s and osmolality of 290–300 mOsm/kg at 37°C. RBC–PVP suspensions are subjected to Couette flow in concentric cylinders or rotational/flow fields in slit channels, with laser diffraction generating EI–SS curves. LORRCA is a representative instrument that combines rotational Couette flow with laser diffraction, whereas RheoScan-D implements the same principle in a microfluidic format. In the steady-state mode of LORRCA, SS is varied across 0.3–30 Pa, yielding EI data from approximately 12 measurement points.

### Syllectometry

Syllectometry quantifies RBC aggregation and involves (i) complete disaggregation of pre-existing RBC aggregates by applying pre-shear, (ii) switching to zero or low shear, and (iii) recording a syllectogram. Time-dependent changes in optical signals, such as transmission or backscattering, immediately after shear cessation are analyzed to quantify RBC aggregation using indices such as the aggregation index (AI; area ratio), *t*_½_ (half-life), AMP (aggregation measurement parameter), and γ (disaggregation threshold), thereby assessing the speed and mechanical stability of aggregation.^9^ To ensure comparability and clinical applicability, the standardization of conditions, including temperature (37°C), pre-shear settings, optical method, gap width, optical window, and area definitions, is critical, along with Hct .

LORRCA derives curves from time-dependent backscattering, whereas Myrenne (Myrenne GmbH) uses transmitted light. According to comparisons at ISCH workshops,^10^ LORRCA, Myrenne, and RheoScan demonstrated sufficient precision for clinical research, each with strengths and limitations under extreme aggregation or very low shear conditions. LORRCA and Myrenne reliably detect aggregation decreases caused by plasma dilution, but Myrenne is less sensitive to subtle RBC stiffening under extremely low shear. RheoScan-A may have difficulty discriminating very strong aggregation.

### Microchannel method

The microchannel method integrates multiple determinants of whole-blood fluidity and microvascular resistance, such as RBC deformability and aggregation, WBC and platelet adhesion and plugging, and plasma viscosity, by measuring whole-blood flow through standardized microchannel arrays at the capillary scale. Key parameters include whole-blood passage time (WBPT), occlusion rate, and adherent leukocyte count, quantified via total flow or passage time and time-lapse imaging under constant pressure.^11–14^

Previously, the filter technique underpinned this approach but suffered from poor pore-density precision because of nucleopore membrane variability^15^ and bubble trapping in pores, reducing reproducibility.^16^ The microchannel method overcomes these limitations, and MC-FANs are used in clinical and epidemiological settings.

**Figure 1:**
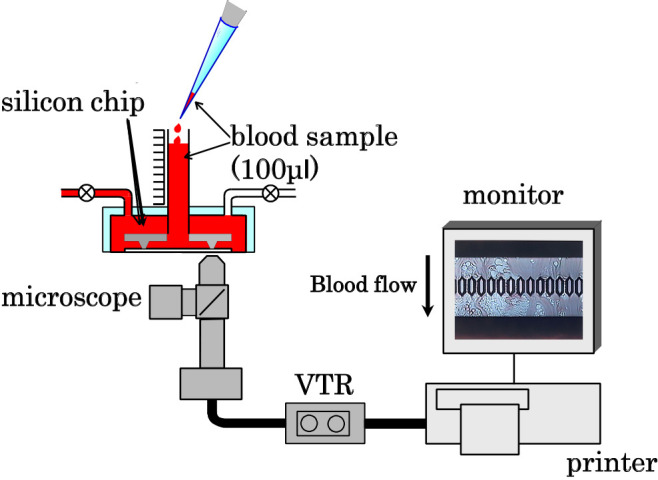
Schematic diagram of MC-FAN

MC-FANs feature thousands of parallel microgrooves, fabricated by photolithography and dry etching, on a silicon chip that is bonded to glass. The analyzer measures the flow rate and passage time under a defined negative pressure. The typical channel dimensions are 7 μm in width, 30 μm in length, and 4.5 μm in depth, with 8,736 channels per chip. A 100-μL blood sample is aspirated using a 20-cm water-column pressure difference, and WBPT, occlusion rate, and leukocyte adhesion are quantified as indices of blood fluidity (Figure 1).

Unlike ektacytometry, which measures physical properties, the microchannel method assesses functional passability and sensitively reflects reduced RBC deformability and microchannel clogging. To control for lot-to-lot variability of silicon chips, saline passage time is measured immediately before each assay for correction. However, the global adoption of MC-FANs remains limited, and standardized protocols and clinical guidelines are lacking.

For standardization, reporting should explicitly include the measurement temperature (e.g., room temperature or 37°C), chip specifications and lot numbers, driving conditions (water-column pressure and flow rate), anticoagulants, sampling-to-measurement interval, and analytic procedures (e.g., 20-μL split time, occlusion rate definitions, and leukocyte counting thresholds). Passage-time correction with saline controls and plasma volume correction using the Dill and Costill method^17^ should be included. The relative contributions of the physical parameters influencing functional passability, measured using the WBPT, also require clarification.

## Diurnal variation in blood fluidity

Epidemiological data show that acute cardiovascular and cerebrovascular events tend to cluster in the early morning hours,^18^ consistent with chronobiological mechanisms, such as increased platelet activation between approximately 06:00 and 12:00 and a simultaneous decrease in the fibrinolytic capacity.^19^ The reduction in cerebral blood flow and diminished vascular reactivity during the night-to-morning transition align with the increased morning stroke risk.^20^ For health promotion, understanding the safest time of day to exercise requires a comprehensive understanding of diurnal variations in blood fluidity.

A study of 24 healthy men using a rotational viscometer repeatedly measured whole-blood viscosity (WBV) across low-to-high shear rates (2–300 s^–1^).^21^ Although limited by its single-center design, short observation period, and issues related to temperature control, Hct correction, posture/meal standardization, and inter-device harmonization, WBV tended to be higher in the morning than in the afternoon, with a slight postprandial increase and greater intra- and inter-individual variability in the morning. Although the mean differences were small and the statistical significance was limited, the authors provided practical sample-size estimations based on power analysis: detecting a 10% change at low shear required 30 samples, whereas at high shear, nine were sufficient, thus guiding the design of intervention and longitudinal studies.

Among the factors contributing to blood fluidity, RBC deformability has been the most thoroughly studied for diurnal variation. Physiological melatonin fluctuations associated with the circadian rhythm influence RBC deformability, and abnormal light–dark cycle exposure may increase cardiovascular risk. In a study using an animal model, male rats were assigned to five groups (12-hour light/12-hour dark (control), constant light, constant darkness, long-day (16-hour light/8-hour dark), and short-day (8-hour light/16-hour dark)), and the EI was measured using laser diffraction. No significant differences were observed between the constant-light and control groups; however, EI was significantly reduced in the constant-dark group (vs. control, *p*=0.009; vs. constant light, *p*=0.05).^22^ EI reduction was observed under long- and short-day conditions (*p*<0.05 and p=0.007, respectively). In obstructive sleep apnea, which is characterized by nocturnal intermittent hypoxia, resting RBC deformability is not markedly altered, but deformability impairment becomes pronounced under oxidative challenge with tert-butyl hydroperoxide. Continuous positive airway pressure therapy attenuates the worsening of RBC aggregation.^23^

Morning deterioration in blood fluidity, which is relevant to physiological thrombosis risk, may result from the combined effects of circadian fluctuations in body temperature and heart rate, along with smaller diurnal variations in Hct, WBC COUNT, and serum lipids. In a small, single-day study involving nine healthy young men, Kimura et al.^24^ examined the determinants of diurnal rhythm in blood fluidity using an MC-FAN. Physical activity was limited (seated or light daily movement), and meal content and timing were standardized to minimize confounding. Physiological parameters, hematological and biochemical tests, and WBPT were measured at six time points from immediately after awakening (07:30) to 21:30. WBPT was longest at 07:30 and shortest at 13:30, showing a negative correlation with body temperature, i.e., an inverse phase relationship with the daytime increase in temperature. Multiple regression analysis identified body temperature, heart rate, Hct, WBC count, and total cholesterol as significant determinants of WBPT, together explaining approximately 67% of its variance (*R^2^*=0.669).

Although these studies provide valuable conceptual insights and suggest coordination among hemorheology, hemodynamics, coagulation, and fibrinolysis, the evidence overall remains limited. Rigorous circadian studies require strict control of behavioral and environmental factors (e.g., sleep–wake schedule, posture, meals, illumination, and temperature), which is costly and demands prolonged participant commitment. Repeated blood sampling imposes a substantial physical burden, and given the relatively small amplitude of diurnal variation in blood fluidity, minor pre-analytical variation may obscure true differences.

## Exercise and changes in blood fluidity

When examining the effects of physical exercise on the human body, it is necessary to recognize that exercise has multiple dimensions.^25^ A single bout of high-intensity exercise induces dehydration and an increase in Hct, leading to elevated blood viscosity and oxidative stress, thereby reducing blood fluidity. In contrast, chronic endurance training expands plasma volume, resulting in hemodilution and, in the long term, improves blood fluidity. The exercise dimensions that require consideration include (i) mode (running, weight/resistance training, cycling, handgrip exercise); (ii) anaerobic exercise (e.g., 100-m sprint) versus endurance exercise (e.g., marathons); (iii) intensity; (iv) environmental conditions; (v) acute versus chronic performance; and (vi) duration and frequency.

### Mode

Localized exercise may influence blood fluidity. For example, in a study examining the effects of forearm handgrip exercise on blood fluidity, the brachial artery shear rate and blood viscosity (ηb) were simultaneously and noninvasively measured during exercise using Doppler ultrasonography and related techniques.^26^ The shear rate was calculated from the blood flow velocity and vessel diameter, and ηb was estimated from this model. When the shear rate increased by approximately 200% during handgrip exercise, ηb paradoxically decreased by 10%–15%. In another study, isometric handgrip strength negatively correlated with RBC aggregability.^27^

Anaerobic exercise affects blood fluidity. In a study evaluating short-term changes in hemorheological variables after a single bout of resistance exercise, the RBC count, hemoglobin (Hb), and Hct increased by 5.6%, 5.4%, and 6.2%, respectively, whereas the plasma volume decreased by 10.1%.^28^ Plasma viscosity increased post-exercise; however, all hemorheological changes were transient and returned to pre-exercise values within 30 minutes.

Regarding the relationship between sports discipline and blood characteristics, a study compared athletes taking part in futsal, sprinting, and triathlons using an exercise test and evaluated RBC count, Hb, Hct, and ΔPV (change in plasma volume).^29^ Beyond 15–18 min of exercise (i.e., from moderate duration onward), all groups showed significant increases in RBC count, Hb concentration, and Hct compared with the resting values. Although the RBC distribution width differed between the groups, the differences in other indices were small, and the pattern of change was similar across disciplines.

### Intensity

To perform exercises safely, it is important to recognize the differential effects of each exercise intensity and incorporate them appropriately. In high-risk groups (e.g., individuals with sickle cell trait), adequate hydration effectively prevents hyperviscosity-related risks,^30^ and maintaining hydration during exercise is likewise important for athletes and healthy individuals.

The impact of exercise intensity on oxidative stress markers was examined during resistance training.^31^ Higher intensities induced greater oxidative stress, as demonstrated by increases in oxidative stress markers (e.g., thiobarbituric acid-reactive substances and protein carbonyls), accompanied by a relatively pronounced decrease in antioxidant capacity. These conditions may indirectly impair RBC membrane rheology by reducing RBC deformability and increasing aggregation.

In a within-subject comparison of acute hemorheological responses to moderate-intensity continuous running versus high-intensity interval training, RBC and WBC counts increased significantly immediately after exercise in the moderate-intensity continuous running condition compared with that during rest.^32^ However, under both conditions, blood volume (BV), plasma volume (PV), and RBC deformability showed no significant changes immediately after exercise, and no clear intensity-dependent differences were observed. These findings were obtained in young, predominantly sedentary men and may not reflect the adaptations associated with habitual training.

A study examining the effect of running distance compared a 40-km trail race with a 171-km ultra-endurance event and reported distance-dependent differences in blood viscosity, RBC aging indices (e.g., phosphatidylserine exposure), and hematological markers.^33^ In ultra-endurance races, intravascular fluid retention and plasma volume re-expansion were observed, indicating protective physiological adaptations that attenuate increases in blood viscosity.

Structural alterations inRBCs after marathon races have also been reported, including a reduction in membrane-associated components and an increase in the erythrocyte membrane skeleton surface area.^34^ These findings suggest that erythrocyte membrane remodeling is associated with prolonged endurance exercise.

Finally, overtraining syndrome (OTS) in athletes has been investigated with respect to blood fluidity. Heavy and fatigued sensations in the legs, a characteristic of OTS, have been suggested to be associated with plasma hyperviscosity, underscoring the potential utility of hemorheological assessment in OTS research. However, plasma viscosity had low sensitivity as a predictive marker for OTS and is not directly useful for diagnosis.^35–37^ Therefore, hemorheological and biochemical indices may serve as complementary indicators for monitoring the condition of athletes, but are insufficient on their own, necessitating further evidence, including other markers.

### Environment

To investigate the effect of increased core temperature during exercise on hemorheology, a study applied aerobic exercise under hot environmental conditions and measured Hct, plasma viscosity (ηp), whole-blood viscosity (ηb), and RBC deformability (EI) . Blood samples obtained before and after exercise were analyzed at different assay temperatures (including 37°C) to evaluate the temperature dependence of blood viscosity.^38^ After approximately 60 minutes of endurance exercise in the heat, Hct and ηp increased modestly; however, EI measured at 37°C improved, such that the rise in ηb was offset and, in some cases, no significant change in ηb was observed.

Endurance runners frequently undergo altitude training to expose themselves to hypoxic conditions. In a study comparing healthy men trained under normoxia or hypoxia, the hypoxic group exhibited greater improvements in cardiopulmonary function than the normoxic group and showed increased RBC aggregability , suggesting a potential trade-off between aerobic adaptation and blood rheology.^39^

### Training status

Post-exercise changes in blood fluidity vary depending on the degree of training and exercise intensity. In athletes, moderate-intensity exercise may induce no change or only transient changes within the reference range; in contrast, in untrained individuals, the effects can persist for more than half a day. Therefore, caution is warranted when prescribing exercise to untrained populations.

In a study of untrained men subjected to high-intensity exercise, RBC deformability and aggregability decreased immediately after exercise, and this reduction persisted for at least 12 hours.^40^ Conversely, a study of 19 male finishers of a 24-hour ultramarathon evaluated RBC viscoelasticity using video particle-tracking micro-rheology before and after the race.^41^ Ultra-long-duration endurance running induced hematological changes similar to those observed in sports anemia, and although no significant changes were detected at the group level, individual variations in membrane viscoelasticity were observed.

In eight male endurance-trained athletes subjected to high-intensity cycling, RBC deformability was reduced, and RBC microparticles (small membrane-derived fragments shed from RBCs) tended to increase post-exercise compared with pre-exercise values.^42^ Concurrent metabolomic and lipidomic remodeling suggested that oxidative stress and membrane repair processes may contribute to the observed decrease in deformability.

In contrast, some reports indicate that blood fluidity does not markedly deteriorate after exercise, and functional improvements other than hyperviscosity can occur under appropriate exercise loading. For example, one study reported improved RBC deformability among athletes, along with reductions in aggregation strength and blood viscosity immediately after a 10-km race.^43^ In a study involving triathletes, although RBC deformability, aggregation, and biochemical markers changed post-competition, all values remained within the reference ranges, suggesting that these changes are transient and reversible.^44^

Finally, the optimization of the physical and mental states to achieve peak performance, i.e., conditioning, is crucial for athletes. During the final pre-competition phase in competitive runners, concurrent assessment of hemorheological and biochemical parameters revealed measurable alterations in blood viscosity and erythrocyte aggregation along with associated biochemical changes. These results suggest that such indices could be applied as supportive diagnostic tools for tracking physiological status across the competitive season.^45^

### Duration and frequency

A classic study from 1987 proposed that decreases in plasma and whole-blood viscosities contribute to the hemorheological improvement seen after regular physical activity.^46^ Regular low- to moderate-intensity exercise tends to improve coagulation and inflammatory profiles and is associated with early reductions in plasma and whole-blood viscosity.

A report on the determinants of blood viscosity stated that endurance exercise and resistance training chronically reduce blood viscosity, primarily through the expansion of plasma volume, over several hours to days following exercise.^47^ Furthermore, because blood exhibits shear-thinning behavior, an increase in intravascular shear rate during exercise leads to a decrease in apparent viscosity, counterbalancing viscosity increases caused by hemoconcentration and reduced RBC deformability. To investigate longer-term adaptation to exercise, a study involving 14 middle-aged men implemented a walking program of 300 kcal/day for 4 weeks.^48^ WBPT was significantly reduced at week 2 compared to baseline, and the improvement was maintained at week 4. Thus, adding approximately 30 minutes of walking daily resulted in a significant reduction (*p*<0.01) in WBPT within 2 weeks, with continued exercise sustaining this effect. The WBPT reduction was further linked to changes in plasma fluidity.

Similarly, among individuals with an established habit of regular jogging, low levels of blood viscosity, plasma viscosity, RBC aggregation, fibrinogen, and hs-CRP (high-sensitivity C-reactive protein), as well as reduced Hct-corrected whole-blood viscosity, were reported, indicating a more favorable internal environment for blood fluidity.^49^

In a cross-sectional comparison of elite athletes from different endurance disciplines,^50^ BV and Hb mass were quantified using the carbon monoxide rebreathing method and calculations based on hematocrit, respectively. In endurance disciplines (running, triathlon, and cycling), BV and Hb were approximately 20% and 10% higher, respectively, than those in the control participants. These findings suggest that long-term endurance training induces volume and oxygen transport adaptations that contribute to stabilizing hemorheology.

The dynamics of exercise and hemorheology have been categorized into short-, medium-, and long-term timescales.^51^ In the short term, immediately after a single bout of exercise, Hct, plasma viscosity, and RBC deformability deteriorate, whereas RBC aggregation and leukocyte activation increase blood viscosity, resulting in hyperviscosity. In the medium term (2–4 weeks), endurance training expands plasma volume by 10%–20%, and improvements in inflammatory and coagulation profiles enhance blood fluidity. In the long term, endurance athletes exhibit increased BV and Hb mass, with stable circulatory adaptations that result in stabilized blood fluidity.

A comprehensive review of oxygen transport by RBCs and the impact of exercise training on RBC number, lifespan, and function highlighted that sports anemia primarily reflects a relative decrease in Hct caused by a 15%–20% increase in plasma volume and should be distinguished from iron-deficiency anemia. Endurance training tends to improve RBC deformability (Maximal Elongation Index +5%–8%), enhancing oxygen transport capacity.^52^

## Utilization of blood fluidity measurement methods

### Appropriate use of devices

Studies on exercise and blood fluidity are summarized by the measurement technique used in Table 2. Each method has specific characteristics, and thus, device selection should align with the primary research question. However, combining multiple methods may often yield more precise results.

Platelets and leukocytes may be activated because of delays between blood sampling and measurement, as well as because of agitation and temperature fluctuations. Hemorheological parameters change most steeply immediately after exercise until approximately 30 minutes post-exercise, making measurement timing and delays critical for accurate results.

A recent report integrating classical evidence with contemporary mechanistic and methodological advances reaffirmed that acute exercise-induced increases in blood viscosity primarily depend on elevated Hct.^53^ Measurements at 25°C tended to overestimate viscosity changes, whereas at 37°C, these changes were attenuated, demonstrating a strong dependence on the sample temperature during measurement. Furthermore, they highlighted that inter-device variability and non-standardized reporting formats (e.g., temperature, shear rate, and Hct correction) contribute to variability and inconsistency in conclusions.

### Analysis of exercise effects using MC-FAN

A study using an MC-FAN to functionally evaluate artificial microvascular network perfusion showed that reduced RBC deformability directly impairs network perfusion.^54^

In a study of 10 healthy young men, participants performed cycling exercises on three separate days at low, moderate, and high intensities.^55^ Under high-intensity conditions, WBPT (100 μL) and split times for each 20-μL increment increased with flow progression, indicating reduced blood fluidity. At the adenosine diphosphate (ADP) threshold level, the transit time increased with platelet aggregation and leukocyte adhesion. When the samples were adjusted to higher Hct values using resting blood, the transit time did not increase as much as after exercise. These findings suggest that post-exercise prolongation of transit time is strongly influenced by microchannel clogging caused by platelet aggregation and leukocyte adhesion and cannot be explained by hemoconcentration alone. In another study involving 10 healthy men subjected to cycling exercise, WBPT, the number of adherent WBCs on microchannels, and the percentage of occluded channels while using an MC-FAN were quantified from video recordings.^56^ After exercise, Hct, WBC count, and WBPT increased significantly (*p*<0.01), whereas LF/HF (low-frequency/high-frequency power ratio in heart rate variability, an index of sympathovagal balance) and nitric oxide (NO) metabolites showed an upward trend. The transient decrease in blood fluidity after high-intensity exercise resulted from increased cell counts and leukocyte activation, contributing to delayed transit (clogginess) in the MC-FAN system.

Another study involving 19 healthy young participants applied 20 minutes of whole-body neuromuscular electrical stimulation as an exercise model to examine the relationship between blood fluidity and sublingual microcirculation.^57^ After stimulation, lactate increased significantly (*p*<0.01) and blood glucose decreased; however, WBPT did not change significantly. In some individuals, WBPT was slightly prolonged immediately after stimulation but returned to baseline within 20 minutes. The sublingual microcirculation remained unchanged. These findings indicate that short, single sessions of electrically induced exercise do not substantially change blood fluidity.

### Increased blood fluidity via NO

Post-exercise prolongation of WBPT may involve the NO pathway, which exerts vasodilatory and platelet inhibitory effects. Immediately after moderate-intensity cycling, phosphorylation of NO synthase (NOS) and NO production in RBCs increased, improving RBC deformability (EI) by 5%–10%.^58^ In vitro inhibition experiments confirmed that these effects were NO-dependent, supporting the role of NO as a regulator of RBC deformability.

NOS inhibition with N^G^-nitro-L-arginine methyl ester or N^G^-monomethyl-L-arginine (L-NMMA) reduces RBC deformability, whereas NO donors (sodium nitroprusside or diethylenetriamine NONOate) increase deformability.^59^ Shear conditioning—in which blood flows faster and more smoothly during daily activities and exercise—restores RBC deformability impaired by superoxide (O_2_^–^) while simultaneously activating RBC-NOS.^60^ Superoxide reduced the phosphorylation of Akt (Ser473), a central kinase involved in shear stress–dependent NOS activation, and NOS (Ser1177) in RBCs, leading to impaired deformability. In contrast, shear conditioning reversed these effects by restoring NOS Ser1177 phosphorylation and improving deformability. These findings indicate that nitric oxide (NO) protects RBCs and that mechanical shear stress activates this Akt–NOS signaling pathway.

In a study on high-intensity exercise and NO, 10 healthy men performed 15 minutes of cycling at 70% VO_2_peak (the highest oxygen uptake measured during maximal exercise).^61^ Blood samples obtained before and after exercise were supplemented with L-arginine or the NOS inhibitor L-NMMA and evaluated using WBPT with an MC-FAN. The occluded channel ratio and the number of adherent WBCs were quantified. After exercise, Hct, WBC, RBC, and platelet count increased, as did the WBPT, occlusion rate, and adherent WBCs. L-arginine reduced WBPT, occlusion rate, and adherent WBCs in a dose-dependent manner, and these improvements were attenuated by L-NMMA. The transit times of suspensions containing RBCs alone were unaffected, indicating that the post-exercise reduction in blood fluidity was primarily caused by platelet aggregation and leukocyte adhesion rather than intrinsic changes in RBCs.

## Assessment of disease activity using MC-FAN

WBPT measured using an MC-FAN has demonstrated clinical utility across multiple domains of clinical practice and preventive medicine, including long-term prospective prediction of cardiovascular events, detection of subclinical myocardial injury, evaluation of endothelial function, assessment of renal microcirculation and arteriosclerosis in patients undergoing hemodialysis, monitoring of sepsis-associated coagulopathy, and evaluation of lifestyle interventions.

WBPT complements established biomarkers, such as the cardio-ankle vascular index (CAVI), high-sensitivity cardiac troponin T, estimated glomerular filtration rate (eGFR) and albumin, and the renal resistive index (RRI). Two-axis evaluation (WBPT×CAVI·hs-cTnT·eGFR+albumin·RRI) combining these markers has repeatedly improved predictive power and diagnostic accuracy in various fields.

In primary prevention, WBPT is especially useful when analyzed with flow-mediated dilation, albuminuria, RRI, and indices of inflammation and arteriosclerosis, serving as an adjunctive diagnostic tool that evaluates the microcirculation through the lens of hemorheological properties. Future efforts should establish standardized WBPT cut-off values, integrate WBPT with other indices such as CAVI, and demonstrate in interventional studies that improving WBPT leads to better clinical outcomes. Developing automated image analysis using machine learning to objectively quantify occlusion and adhesion events and numerically ensure inter-device and inter-center reproducibility will be essential for advancing multicenter collaborative studies.

### Disordered glucose metabolism

Blood fluidity may be impaired in the early stages of metabolic abnormalities. In 151 healthy young adults (mean age 24 years), the homeostasis model assessment of insulin resistance correlated positively with WBPT, and Hct, fibrinogen, WBC count, and lipids were identified as determinants of WBPT.^62^ Advanced glycation end products (AGEs) are compounds formed when sugars non-enzymatically bind to proteins or lipids, and their formation is accelerated by hyperglycemia in diabetes. AGE accumulation in tissues accelerates aging and chronic diseases. The relationship between AGEs and blood fluidity has been discussed in several studies.^63–65^ Accumulation of AGEs induces endothelial activation, which promotes leukocyte adhesion and platelet aggregation within the vasculature. These processes increase the propensity for microvascular obstruction, thereby impairing microcirculatory blood flow and resulting in the prolongation of WBPT.

### Renal impairment

In an analysis of 453 patients with hypertension, WBPT was negatively correlated with eGFR and positively correlated with urinary albumin excretion (*r*=0.40) and RRI.^66^ In the multivariate analysis, WBPT was the dependent variable, and albuminuria, RRI, and oxidative stress markers emerged as independent explanatory variables.

In patients undergoing dialysis, WBPT serves as an index of arteriosclerosis and inflammation. In 118 patients undergoing hemodialysis, WBPT was measured using an MC-FAN and correlated strongly with fibrinogen and inflammatory markers such as hs-CRP, intima-media thickness, and brachial-ankle pulse wave velocity (baPWV). In a multivariate analysis, baPWV, intima-media thickness, fibrinogen, Hct, WBC count, and hs-CRP were identified as determinants of WBPT.^67^

### Cardiovascular disease

Impaired blood rheology reflects an increased risk of major cardiovascular events and is associated with endothelial dysfunction. In a cohort of outpatients with cardiovascular risk factors, those with WBPT ≥70 s had a higher incidence of major cardiovascular events than those with WBPT <50 s, and the optimal WBPT cut-off was 72.4 s. When combined with a CAVI ≥9, the hazard ratio increased to 10.62 for major cardiovascular events, such as myocardial infarction.^68^ Serum high-sensitivity cardiac troponin T is a risk factor for cardiovascular diseases, and its elevation is associated with a tendency toward prolonged WBPT in outpatients with type 2 diabetes mellitus.^69^

In a cohort of patients with coronary risk factors, WBPT was negatively correlated with brachial flow-mediated dilation, indicating that impaired blood rheology is associated with endothelial dysfunction.^70^

### Sepsis

Leukocyte rheology assessed using an MC-FAN has been proposed as a candidate bedside index related to microcirculatory failure and disease severity in patients with sepsis. In cases of sepsis-associated coagulopathy, polymorphonuclear neutrophil deformability measured using the MC-FAN system correlated with the Japanese Association for Acute Medicine disseminated intravascular coagulation score and treatment responsiveness.^71^

## Effects of nutritional interventions and lifestyle factors on blood fluidity

Nutritional interventions that may improve blood fluidity include L-arginine, fish oil, astaxanthin, and L-cysteine. In addition, dietary components that are common in Japan, such as dried bonito broth and tea constituents, have been investigated (Table 3). Acetaldehyde (ACD), a metabolite produced during alcohol metabolism, has been examined as a factor that may impair blood fluidity. However, the dose, timing (pre- versus post-exercise), physiological concentration range, mechanistic dissection, and external validity in human studies of nutritional interventions have not yet been sufficiently evaluated.

For analyzing the effects of nutritional interventions on blood fluidity, the MC-FAN system has a unique advantage over other analytical methods in that it can visualize the impact of such interventions on WBPT, microchannel occlusion rate, and leukocyte adhesion to microchannels.

### Tea constituents

Studies on tea constituents commonly consumed in Japan include ingestion and additive experiments. For example, a single ingestion of 250 mL barley tea (mugicha) significantly shortened WBPT at 1 hour, and in vitro addition of the volatile aroma component of tea 2,3,5-trimethylpyrazine shortened passage time.^72^

### L- arginine

In healthy men subjected to high-intensity cycling, L-arginine-derived NO improved RBC deformability and reduced the WBPT. Exercise-induced deterioration of blood fluidity, including increased WBPT, occluded-channel ratio, and leukocyte adhesion number, was observed using an MC-FAN. However, supplementation with the NO precursor L-arginine shortened the WBPT in a dose-dependent manner and reduced leukocyte adhesion and occlusion, whereas the NOS inhibitor L-NMMA abolished these effects. These findings suggest that exercise-induced impairment of blood fluidity, accompanied by increased platelet aggregation and leukocyte adhesion, can be ameliorated via the L-arginine–NO pathway.^61^

In a hypercholesterolemia model, the addition of an NO donor, a cyclic guanosine monophosphate analog, or L-arginine improved reduced RBC deformability and decreased WBPT measured by MC-FAN.^73^

Although preclinical, these findings suggest that activation of the NO pathway could improve RBC deformability, reducing microcirculatory resistance.

### Fish oil

MC-FAN analysis showed that there was minimal effect when rats were fed fish oil (n-3 polyunsaturated fatty acids, primarily eicosapentaenoic acid and docosahexaenoic acid) on RBC deformability, but a shortening of WBPT was observed.^74^ In contrast, in the corn oil group, frequent microaggregates of platelets were observed. These findings indicate that fish oil improves blood fluidity and suggest that its primary site of action is more in the suppression of platelet aggregation than in the enhancement of RBC membrane deformability.

### Astaxanthin

Astaxanthin is a naturally occurring carotenoid with strong antioxidant properties. In a placebo-controlled study of healthy adults, oral supplementation with astaxanthin (6 mg/day for 10 days) significantly shortened whole blood passage time (WBPT) assessed by a microchannel array flow analyzer compared with placebo (p<0.05).^75^ These findings indicate that short-term astaxanthin supplementation can improve blood fluidity under resting conditions in healthy individuals.

### Dried bonito broth

Using MC-FAN analysis, habitual consumption of dried bonito broth was shown to shorten WBPT and simultaneously reduce oxidative stress markers.^76^

Together, these findings demonstrate that food-derived components can improve whole-blood fluidity in microchannels and suggest that daily dietary interventions may contribute to improved blood fluidity.

### Alcohol

In a study of the general adult population, drinking habits were independently and inversely associated with WBPT, which was measured using an MC-FAN, suggesting that light-to-moderate alcohol consumption may improve blood rheology.^77^ Conversely, acetaldehyde, a metabolite produced during alcohol metabolism, is a direct and deleterious factor for blood fluidity, and its effects are attenuated by L-cysteine.^78^ MC-FAN analysis showed that acetaldehyde worsened blood fluidity in a concentration-dependent manner, significantly prolonging the WBPT and increasing the occluded-channel rate and leukocyte adhesion to microchannels.

### Smoking

A study involving 74 smokers demonstrated that higher smoking intensity was associated with prolonged WBPT, and a 3-month smoking cessation period resulted in a significant improvement in WBPT (*p*=0.002).^79^ These findings indicate a close relationship between cigarette smoking and impaired blood rheology.

## Conclusion

MC-FAN uniquely quantifies the functional clogging tendency of the microcirculation, enabling the visualization of the combined exercise and nutritional effects by monitoring whole-blood passage through capillary-mimicking microchannels. Without sample pre-processing, it evaluates the functional passage capacity (WBPT, occluded-channel rate, and adherent WBCs). The resulting measurements integrate the effects of RBC deformability and aggregation, WBC adhesion, platelet microthrombi, plasma viscosity, and Hct, reflecting a state closer to in vivo conditions. The standardization of measurement methodologies remains challenging. Systems capable of disentangling and quantifying the respective contributions of hemoconcentration/hemodilution and cell activation-induced microchannel occlusion are needed. With the MC-FAN system as a core tool, integrative research linking basic material properties, functional capacity, and clinical outcomes is expected to drive breakthroughs in exercise safety and primary cardiovascular prevention.

## Figures and Tables

**Table 1:** Devices used to analyze blood fluidity

Device category	Examples of Instruments	Primary sample type	Measurement targets
Cone–plate viscometry (rotational rheometer)	A rotational viscometer (Brookfield)	Whole blood, plasma, or RBC resuspensions	Apparent viscosity
Ektacytometry	LORRCA (RR Mechatronics) RheoScan-D (RheoMeditech)	RBC–PVP suspension (isotonic, high-viscosity medium)	RBC deformability
Syllectometry	Myrenne (Myrenne GmbH) LORRCA (RR Mechatronics) RheoScan-A (RheoMeditech)	Whole blood (Hct standardization recommended) or RBC suspensions	RBC aggregation
Microchannel methods	MC-FAN (OPTIMA Inc)	Whole blood with anticoagulant	Whole blood Fluidity

**Table 2:** Characteristics and challenges of measurement methods

Measurement method	Characteristics	Challenges
Viscosity methods	Captures overall hemodynamic load in a single value; intuitive for trend tracking	Strong dependence on temperature, hematocrit, and shear rate; may diverge from microcirculatory behavior
Ektacytometry	Quantifies shear-dependent red blood cell deformability; useful as a mechanistic index for acute loading and training adaptations	Bias from polyvinylpyrrolidone viscosity/molecular weight, temperature, device-to-device differences, and pre-processing, e.g., pre-shear, anticoagulant, and storage
Syllectometry	Assesses low-shear red blood cell aggregation dynamics	Extremely sensitive to hematocrit and fibrinogen; pre-shear dependent
Microchannel methods	Reflects effective microcirculatory bottlenecks; can help predict cardiovascular events	Strong effects of chip geometry/lot, temperature, and driving pressure; difficult separation mechanism

**Table 3:** The effect of nutrition on blood fluidity

Nutrient	Primary mechanisms	Effect
Barley tea (mugicha)	Suppression of excessive platelet activation and oxidative damage to the erythrocyte membrane	Decreased whole blood passage time (WBPT)
L-Arginine	NO pathway activation; reduces White blood cell adhesion (WBC) and microvascular occlusion	Decreased WBPT, occlusion, and WBC adhesion in post-exercise samples; effect blocked by L-NMMA
Fish oil (Eicosapentaenoic acid / Docosahexaenoic acid)	Platelet micro-aggregation inhibition; membrane incorporation; anti-inflammatory effects	Suggests improvement of microvascular occlusion risk via platelet inhibition
Astaxanthin	Antioxidant; lowers red blood cell membrane lipid peroxidation	Decreased WBPT
Dried bonito broth (Katsuodashi)	Oxidative stress reduction; microcirculatory facilitation	Decreased WBPT; increased peripheral blood flow and decreased urinary 8-OHdG reported in adjunct studies
Acetaldehyde±L-cysteine	Acetaldehyde impairs rheology; L-cysteine scavenges and neutralizes aldehyde	Acetaldehyde increases WBPT, microvascular occlusion and WBC adhesion; co-treatment with L-cysteine attenuates changes

## References

[1] Fåhræus R, Lindqvist T. The viscosity of the blood in narrow capillary tubes. American Journal of Physiology 1931; 96: 562–568.

[2] Fåhræus R. The suspension stability of the blood. Physiol Rev 1929; 9: 241–274.

[3] Baskurt OK, Boynard M, Cokelet GC, et al. New guidelines for hemorheological laboratory techniques. Clin Hemorheol Microcirc 2009; 42: 75–97. 19433882 10.3233/CH-2009-1202

[4] Oh JS, Prabhakaran P, Seo DK, Kim DY, Lee W, Ahn KH. A comparative study of blood viscometers of 3 different types. Clin Hemorheol Microcirc 2024; 88: 211–219. 38905037 10.3233/CH-242256

[5] Ihm C, Lee D-S, Ahn KH, Oh JS. Viscosity Measurement of Whole Blood with Parallel Plate Rheometers. Biochip J 2020; 14: 179–184.

[6] Shin S, Ku Y, Park MS, Suh JS. Measurement of blood viscosity using a pressure-scanning capillary viscometer. Clin Hemorheol Microcirc 2004; 30: 467–470. 15258389

[7] Wells RE Jr, Denton R, Merrill EW. Measurement of viscosity of biologic fluids by cone plate viscometer. J Lab Clin Med 1961; 57: 646–656. 13784261

[8] Hardeman MR, Dobbe JG, Ince C. The Laser-assisted Optical Rotational Cell Analyzer (LORCA) as red blood cell aggregometer. Clin Hemorheol Microcirc 2001; 25: 1–11. 11790865

[9] Dobbe JG, Streekstra GJ, Strackee J, Rutten MC, Stijnen JM, Grimbergen CA. Syllectometry: the effect of aggregometer geometry in the assessment of red blood cell shape recovery and aggregation. IEEE Trans Biomed Eng 2003; 50: 97–106. 12617529 10.1109/TBME.2002.807319

[10] Baskurt OK, Uyuklu M, Ulker P, Cengiz M, Nemeth N, Alexy T, Shin S, Hardeman MR, Meiselman HJ. Comparison of three instruments for measuring red blood cell aggregation. Clin Hemorheol Microcirc 2009; 43: 283–298. 19996518 10.3233/CH-2009-1240

[11] Kikuchi Y. Effect of leukocytes and platelets on blood flow through a parallel array of microchannels: micro- and macroflow relation and rheological measures of leukocyte and platelet activities. Microvasc Res 1995; 50: 288–300. 8538506 10.1006/mvre.1995.1059

[12] Kikuchi Y, Da QW, Fujino T. Variation in red blood cell deformability and possible consequences for oxygen transport to tissue. Microvasc Res 1994; 47: 222–231. 8022320 10.1006/mvre.1994.1017

[13] Kikuchi Y, Sato K, Mizuguchi Y. Modified cell-flow microchannels in a single-crystal silicon substrate and flow behavior of blood cells. Microvasc Res 1994; 47: 126–139. 8022310 10.1006/mvre.1994.1008

[14] Kikuchi Y, Sato K, Ohki H, Kaneko T. Optically accessible microchannels formed in a single-crystal silicon substrate for studies of blood rheology. Microvasc Res 1992; 44: 226–240. 1474929 10.1016/0026-2862(92)90082-z

[15] Kikuchi Y, Koyama T. Role of protein adsorption in micropore passability of red blood cells. Jpn J Physiol 1981; 31: 903–915. 7341816 10.2170/jjphysiol.31.903

[16] Kikuchi Y, Horimoto M, Koyama T, Koyama Y, Tozawa S. Estimation of pore passage time of red blood cells in normal subjects and patients with renal failure. Experientia 1980; 36: 325–327. 7371789 10.1007/BF01952304

[17] Dill DB, Costill DL. Calculation of percentage changes in volumes of blood, plasma, and red cells in dehydration. J Appl Physiol 1974; 37: 247–248. 4850854 10.1152/jappl.1974.37.2.247

[18] Cohen MC, Rohtla KM, Lavery CE, Muller JE, Mittleman MA. Meta-analysis of the morning excess of acute myocardial infarction and sudden cardiac death. Am J Cardiol 1997; 79: 1512–1516. 9185643 10.1016/s0002-9149(97)00181-1

[19] Scheer FA, Michelson AD, Frelinger AL 3rd, Evoniuk H, Kelly EE, McCarthy M, Doamekpor LA, Barnard MR, Shea SA. The human endogenous circadian system causes greatest platelet activation during the biological morning independent of behaviors. PLoS One 2011; 6: e24549. 21931750 10.1371/journal.pone.0024549 PMC3169622

[20] Elliott WJ. Circadian variation in the timing of stroke onset: a meta-analysis. Stroke 1998; 29: 992–996. 9596248 10.1161/01.str.29.5.992

[21] Wang S, Boss AH, Kensey KR, Rosenson RS. Variations of whole blood viscosity using Rheolog-a new scanning capillary viscometer. Clin Chim Acta 2003; 332: 79–82. 12763283 10.1016/s0009-8981(03)00125-6

[22] Yerer MB, Aydoğan S. The importance of circadian rhythm alterations in erythrocyte deformability. Clin Hemorheol Microcirc 2006; 35: 143–147. 16899919

[23] Waltz X, Beaudin AE, Belaidi E, Raneri J, Pépin JL, Pialoux V, Hanly PJ, Verges S, Poulin MJ. Impact of obstructive sleep apnoea and intermittent hypoxia on blood rheology: a translational study. Eur Respir J 2021; 58: 2100352. 33863746 10.1183/13993003.00352-2021

[24] Kimura T, Inamizu T, Sekikawa K, Kakehashi M, Onari K. Determinants of the daily rhythm of blood fluidity. J Circadian Rhythms 2009; 7: 7. 19558641 10.1186/1740-3391-7-7 PMC2711049

[25] Jakicic JM, Apovian CM, Barr-Anderson DJ, Courcoulas AP, Donnelly JE, Ekkekakis P, Hopkins M, Lambert EV, Napolitano MA, Volpe SL. Physical Activity and Excess Body Weight and Adiposity for Adults. American College of Sports Medicine Consensus Statement. Med Sci Sports Exerc 2024; 56: 2076–2091. 39277776 10.1249/MSS.0000000000003520

[26] Leo JA, Simmonds MJ, Sabapathy S. Shear-thinning behaviour of blood in response to active hyperaemia: Implications for the assessment of arterial shear stress-mediated dilatation. Exp Physiol 2020; 105: 244–257. 31713290 10.1113/EP088226

[27] Brun JF, Varlet-Marie E, Cassan D, Raynaud de Mauverger E. Blood rheology and body composition as determinants of exercise performance in female rugby players. Clin Hemorheol Microcirc 2011; 49: 207–214. 22214691 10.3233/CH-2011-1470

[28] Ahmadizad S, El-Sayed MS. The acute effects of resistance exercise on the main determinants of blood rheology. J Sports Sci 2005; 23: 243–249. 15966342 10.1080/02640410410001730151

[29] Ciekot-Sołtysiak M, Kusy K, Podgórski T, Pospieszna B, Zieliński J. Changes in red blood cell parameters during incremental exercise in highly trained athletes of different sport specializations. PeerJ 2024; 12: e17040. 38560450 10.7717/peerj.17040 PMC10981411

[30] Tripette J, Loko G, Samb A, Gogh BD, Sewade E, Seck D, Hue O, Romana M, Diop S, Diaw M, Brudey K, Bogui P, Cissé F, Hardy-Dessources MD, Connes P. Effects of hydration and dehydration on blood rheology in sickle cell trait carriers during exercise. Am J Physiol Heart Circ Physiol 2010; 299: H908–H914. 20581085 10.1152/ajpheart.00298.2010

[31] Cakir-Atabek H, Demir S, PinarbaŞili RD, Gündüz N. Effects of different resistance training intensity on indices of oxidative stress. J Strength Cond Res 2010; 24: 2491–2497. 20802287 10.1519/JSC.0b013e3181ddb111

[32] Findikoglu G, Kilic-Toprak E, Kilic-Erkek O, Senol H, Bor-Kucukatay M. Acute effects of continuous and intermittent aerobic exercises on hemorheological parameters: a pilot study. Biorheology 2014; 51: 293–303. 25480930 10.3233/BIR-14012

[33] Robert M, Stauffer E, Nader E, Skinner S, Boisson C, Cibiel A, Feasson L, Renoux C, Robach P, Joly P, Millet GY, Connes P. Impact of Trail Running Races on Blood Viscosity and Its Determinants: Effects of Distance. Int J Mol Sci 2020; 21: 8531. 33198320 10.3390/ijms21228531 PMC7696476

[34] Jordan J, Kiernan W, Merker HJ, Wenzel M, Beneke R. Red cell membrane skeletal changes in marathon runners. Int J Sports Med 1998; 19: 16–19. 9506794 10.1055/s-2007-971873

[35] Varlet-Marie E, Gaudard A, Mercier J, Bressolle F, Brun JF. Is the feeling of heavy legs in overtrained athletes related to impaired hemorheology? Clin Hemorheol Microcirc 2003; 28: 151–159. 12775897

[36] Varlet-Marie E, Maso F, Lac G, Brun JF. Hemorheological disturbances in the overtraining syndrome. Clin Hemorheol Microcirc 2004; 30: 211–218. 15258345

[37] Varlet-Marie E, Mercier J, Brun JF. Is plasma viscosity a predictor of overtraining in athletes? Clin Hemorheol Microcirc 2006; 35: 329–332. 16899952

[38] Buono MJ, Krippes T, Kolkhorst FW, Williams AT, Cabrales P. Increases in core temperature counterbalance effects of haemoconcentration on blood viscosity during prolonged exercise in the heat. Exp Physiol 2016; 101: 332–342. 26682653 10.1113/EP085504 PMC4738148

[39] Lin C-L, Wang J-S, Fu T-C, Hsu C-C, Huang Y-C. Hypoxic Exercise Training Elevates Erythrocyte Aggregation. Applied Sciences 2021; 11: 6038.

[40] Yalcin O, Erman A, Muratli S, Bor-Kucukatay M, Baskurt OK. Time course of hemorheological alterations after heavy anaerobic exercise in untrained human subjects. J Appl Physiol 2003; 94: 997–1002. 12391137 10.1152/japplphysiol.00368.2002

[41] Liu CH, Tseng YF, Lai JI, Chen YQ, Wang SH, Kao WF, Li LH, Chiu YH, How CK, Chang WH. The changes of red blood cell viscoelasticity and sports anemia in male 24-hr ultra-marathoners. J Chin Med Assoc 2018; 81: 475–481. 29133160 10.1016/j.jcma.2017.09.011

[42] Nemkov T, Skinner SC, Nader E, Stefanoni D, Robert M, Cendali F, Stauffer E, Cibiel A, Boisson C, Connes P, D’Alessandro A. Acute Cycling Exercise Induces Changes in Red Blood Cell Deformability and Membrane Lipid Remodeling. Int J Mol Sci 2021; 22: 896. 33477427 10.3390/ijms22020896 PMC7831009

[43] Szpernalowska A, Marcinkowska-Gapińska A. Influence of physical activity on the values of hemorheological parameters. Quality in Sport 2024; 22: 54476.

[44] Teleglow A, Marchewka J, Tota L, Mucha D, Ptaszek B, Makuch R, Mucha D. Changes in blood rheological properties and biochemical markers after participation in the XTERRA Poland triathlon competition. Sci Rep 2022; 12: 3349. 35232974 10.1038/s41598-022-07240-1 PMC8888667

[45] Teległów A, Mirek W, Sudoł G, Podsiadło S, Rembiasz K, Ptaszek B. Rheological and Biochemical Properties of Blood in Runners: A Preliminary Report. Applied Sciences 2024; 14.

[46] Ernst E. Influence of regular physical activity on blood rheology. Eur Heart J 1987; 8 Suppl G: 59–62. 10.1093/eurheartj/8.suppl_g.59 3443127

[47] Nader E, Skinner S, Romana M, Fort R, Lemonne N, Guillot N, Gauthier A, Antoine-Jonville S, Renoux C, Hardy-Dessources MD, Stauffer E, Joly P, Bertrand Y, Connes P. Blood Rheology: Key Parameters, Impact on Blood Flow, Role in Sickle Cell Disease and Effects of Exercise. Front Physiol 2019; 10: 1329. 31749708 10.3389/fphys.2019.01329 PMC6842957

[48] Kimura T, Kawasaki K, Onari K. The effect of walking exercise on hemorheological parameters. Hemorheology and Related Research 2003; 6: 89–98 (in Japanese).

[49] Wu YF, Chen JL, Tsai CS, Hong WE, Hsu PS. Effects of regular exercise on blood rheology. Sci Rep 2025; 15: 26128. 40681539 10.1038/s41598-025-08337-z PMC12274591

[50] Heinicke K, Wolfarth B, Winchenbach P, Biermann B, Schmid A, Huber G, Friedmann B, Schmidt W. Blood volume and hemoglobin mass in elite athletes of different disciplines. Int J Sports Med 2001; 22: 504–512. 11590477 10.1055/s-2001-17613

[51] Brun JF. Exercise hemorheology as a three acts play with metabolic actors: is it of clinical relevance? Clin Hemorheol Microcirc 2002; 26: 155–174. 12082247

[52] Mairbaurl H. Red blood cells in sports: effects of exercise and training on oxygen supply by red blood cells. Front Physiol 2013; 4: 332. 24273518 10.3389/fphys.2013.00332 PMC3824146

[53] Connes P, Pichon A, Hardy-Dessources MD, Waltz X, Lamarre Y, Simmonds MJ, Tripette J. Blood viscosity and hemodynamics during exercise. Clin Hemorheol Microcirc 2012; 51: 101–109. 22240371 10.3233/CH-2011-1515

[54] Shevkoplyas SS, Yoshida T, Gifford SC, Bitensky MW. Direct measurement of the impact of impaired erythrocyte deformability on microvascular network perfusion in a microfluidic device. Lab Chip 2006; 6: 914–920. 16804596 10.1039/b601554a

[55] Kimura T, Hamada H, Taito S, Takahashi M, Sekikawa K. The effect of exercise on blood fluidity: Use of the capillary model to assess the clogginess of blood. Clin Hemorheol Microcirc 2016; 61: 559–569. 25267457 10.3233/CH-141893

[56] Ikeda N, Yasu T, Tsuboi K, Sugawara Y, Kubo N, Umemoto T, Arao K, Kawakami M, Momomura S. Effects of submaximal exercise on blood rheology and sympathetic nerve activity. Circ J 2010; 74: 730–734. 20190425 10.1253/circj.cj-09-0758

[57] Hoshiai M, Ochiai K, Tamura Y, et al. Effects of whole-body neuromuscular electrical stimulation device on hemodynamics, arrhythmia, and sublingual microcirculation. Heart Vessels 2021; 36: 844–852. 33547929 10.1007/s00380-020-01755-1 PMC8093154

[58] Suhr F, Brenig J, Muller R, Behrens H, Bloch W, Grau M. Moderate exercise promotes human RBC-NOS activity, NO production and deformability through Akt kinase pathway. PLoS One 2012; 7: e45982. 23049912 10.1371/journal.pone.0045982 PMC3457942

[59] Bor-Kucukatay M, Wenby RB, Meiselman HJ, Baskurt OK. Effects of nitric oxide on red blood cell deformability. Am J Physiol Heart Circ Physiol 2003; 284: H1577–H1584. 12521942 10.1152/ajpheart.00665.2002

[60] Kuck L, Grau M, Bloch W, Simmonds MJ. Shear Stress Ameliorates Superoxide Impairment to Erythrocyte Deformability With Concurrent Nitric Oxide Synthase Activation. Front Physiol 2019; 10: 36. 30804795 10.3389/fphys.2019.00036 PMC6370721

[61] Namba H, Hamada H, Kimura T, Sekikawa K, Kamikawa N, Ishio-Ueoka H, Kajiwara T, Sato YM, Aizawa F, Yoshida T. Effects of L-arginine on impaired blood fluidity after high-intensity exercise: An in vitro evaluation. Clin Hemorheol Microcirc 2022; 82: 1–12. 35599472 10.3233/CH-211201 PMC9741740

[62] Yoshida K, Kimura T, Aoki T, Tsunekawa K, Araki O, Shoho Y, Nara M, Sumino H, Murakami M. Fasting serum insulin levels and insulin resistance are associated with blood rheology in Japanese young adults without diabetes. J Int Med Res 2016; 44: 496–507. 26920928 10.1177/0300060515627561 PMC5536708

[63] Morigi M, Angioletti S, Imberti B, Donadelli R, Micheletti G, Figliuzzi M, Remuzzi A, Zoja C, Remuzzi G. Leukocyte-endothelial interaction is augmented by high glucose concentrations and hyperglycemia in a NF-kB-dependent fashion. J Clin Invest 1998; 101: 1905–1915. 9576755 10.1172/JCI656 PMC508777

[64] Hasegawa Y, Suehiro A, Higasa S, Namba M, Kakishita E. Enhancing effect of advanced glycation end products on serotonin-induced platelet aggregation in patients with diabetes mellitus. Thromb Res 2002; 107: 319–323. 12565718 10.1016/s0049-3848(02)00348-1

[65] Hitsumoto T. Relationship between cardiovascular risk factors and hemorheology assessed by microchannel method in patients with type 2 diabetes mellitus. Diabetol Int 2017; 8: 316–322. 30603337 10.1007/s13340-017-0314-2 PMC6224886

[66] Hitsumoto T. Relationship Between Hemorheology Assessed Using Microchannel Array Flow Analyzer and Kidney Function in Hypertensive Patients. Cardiol Res 2017; 8: 147–153. 28868099 10.14740/cr572w PMC5574286

[67] Kobayashi S, Okamoto K, Maesato K, Moriya H, Ohtake T. Important role of blood rheology in atherosclerosis of patients with hemodialysis. Hemodial Int 2005; 9: 268–274. 16191077 10.1111/j.1492-7535.2005.01141.x

[68] Hitsumoto T. Usefulness of the Whole Blood Passage Time as a Predictor of Primary Cardiovascular Events in Patients With Traditional Cardiovascular Risk Factors. Cardiol Res 2018; 9: 231–238. 30116451 10.14740/cr763w PMC6089470

[69] Hitsumoto T. Association of Hemorheology With High-Sensitivity Cardiac Troponin T Levels in Patients With Type 2 Diabetes Mellitus Assessed by Microchannel Array Flow Analyzer. Cardiol Res 2017; 8: 304–311. 29317973 10.14740/cr632w PMC5755662

[70] Yagi H, Sumino H, Aoki T, Tsunekawa K, Araki O, Kimura T, Nara M, Ogiwara T, Murakami M. Impaired blood rheology is associated with endothelial dysfunction in patients with coronary risk factors. Clin Hemorheol Microcirc 2016; 62: 139–150. 26444592 10.3233/CH-151960 PMC4927888

[71] Morikawa M, Inoue Y, Sumi Y, Kuroda Y, Tanaka H. Leukocyte deformability is a novel biomarker to reflect sepsis-induced disseminated intravascular coagulation. Acute Med Surg 2015; 2: 13–20. 29123685 10.1002/ams2.54 PMC5667187

[72] Suganuma H, Inakuma T, Kikuchi Y. Amelioratory effect of barley tea drinking on blood fluidity. J Nutr Sci Vitaminol (Tokyo) 2002; 48: 165–168. 12171439 10.3177/jnsv.48.165

[73] Kuwai T, Hayashi J. Nitric oxide pathway activation and impaired red blood cell deformability with hypercholesterolemia. J Atheroscler Thromb 2006; 13: 286–294. 17192693 10.5551/jat.13.286

[74] Watanabe N, Funayama K, Kimura F, Endo Y, Fujimoto K, Kikuchi Y. Effect of Dietary Supplementation of n-3 Polyunsaturated Fatty Acids (PUFA) on Red Blood Cell Deformability and Blood Viscosity in Rats. Journal of Oleo Science 2003; 52: 397–406.

[75] Miyawaki H, Takahashi J, Tsukahara H, Takehara I. Effects of astaxanthin on human blood rheology. J Clin Biochem Nutr 2008; 43: 69–74. 18818755 10.3164/jcbn.2008048 PMC2533721

[76] Nozawa Y, Ishizaki T, Kuroda M, Takahashi K, Ebihara S, Itoh T. Ingestion of Dried-bonito Broth Ameliorates Blood Fluidity in Humans. Journal of Health Science 2007; 53: 543–551.

[77] Kotani K, Adachi S, Osaki Y, Kurozawa Y, Araga S. Relationship between alcohol habits and hemorheology by a micro channel method in a general population. Clin Cardiol 2008; 31: 488–491. 18855354 10.1002/clc.20275 PMC6653150

[78] Otoyama I, Hamada H, Kimura T, Namba H, Sekikawa K, Kamikawa N, Kajiwara T, Aizawa F, Sato YM. L-cysteine improves blood fluidity impaired by acetaldehyde: In vitro evaluation. PLoS One 2019; 14: e0214585. 30925182 10.1371/journal.pone.0214585 PMC6440629

[79] Shimada S, Hasegawa K, Wada H, Terashima S, Satoh-Asahara N, Yamakage H, Kitaoka S, Akao M, Shimatsu A, Takahashi Y. High blood viscosity is closely associated with cigarette smoking and markedly reduced by smoking cessation. Circ J 2011; 75: 185–189. 21071876 10.1253/circj.cj-10-0335

